# Effects of 28-day simvastatin administration on emotional processing, reward learning, working memory, and salivary cortisol in healthy participants at-risk for depression: OxSTEP, an online experimental medicine trial

**DOI:** 10.1017/S0033291725001187

**Published:** 2025-05-22

**Authors:** Riccardo De Giorgi, Shona Waters, Amy L. Gillespie, Alice M. G. Quinton, Michael J. Colwell, Susannah E. Murphy, Philip J. Cowen, Catherine J. Harmer

**Affiliations:** 1 Department of Psychiatry, Warneford Hospital, University of Oxford, Oxford, UK; 2 Oxford Health NHS Foundation Trust, Warneford Hospital, Oxford, UK; 3 Social, Genetic & Developmental Psychiatry Centre, Institute of Psychiatry, Psychology and Neuroscience, King’s College London, London, UK

**Keywords:** at-risk for depression, emotional processing, loneliness, online experimental medicine study, reward learning, simvastatin, waking salivary cortisol, working memory

## Abstract

**Background:**

Statins are among the most prescribed medications worldwide. Both beneficial (e.g. antidepressant and pro-cognitive) and adverse (e.g. depressogenic and cognitive-impairing) mental health outcomes have been described in clinical studies. The underlying neuropsychological mechanisms, whether positive or negative, are, however, not established. Clarifying such activities has implications for the safe prescribing and repurposing potential of these drugs, especially in people with depression.

**Methods:**

In this double-blind, randomized, placebo-controlled experimental medicine study, we investigated the effects of simvastatin on emotional processing, reward learning, working memory, and waking salivary cortisol (WSC) in 101 people at-risk for depression due to reported high loneliness scores (mean 7.3 ± 1.2 on the UCLA scale). This trial was largely conducted during periods of social distancing due to the COVID-19 pandemic (July 2021–February 2023), and we employed a fully remote design within a UK-wide sample.

**Results:**

High retention rates, minimal outlier data, and typical main effects of task condition (e.g. emotion) were seen in all cognitive tasks, indicating this approach was comparable to in-person testing. After 28 days, we found no statistically significant differences (F’s < 3.0, p’s > 0.20) for any of the measures of emotional processing, reward learning, working memory, and WSC.

**Conclusions:**

Study results do not substantiate concerns regarding adverse neuropsychiatric events due to statins and support the safety of their prescribing in at-risk populations. Although other unmeasured cognitive processes may be involved, our null findings are also in line with more recent clinical evidence suggesting statins do not show antidepressant or pro-cognitive efficacy.

## Introduction

Statins are medications that lower peripheral cholesterol by inhibiting the liver enzymes 3-hydroxy-3-methylglutaryl-coenzyme A (HMG-CoA) reductase (Endo, Kuroda, & Tanzawa, 1976), thus decreasing cardiometabolic morbidity and mortality (Sizar, Khare, Patel, & Talati, 2024). These drugs also express a broader pharmacological activity on neurobiological and immune-metabolic processes likely involved in depression pathophysiology (De Giorgi et al., 2023; Jones et al., 2021; Walker et al., 2021). However, the direction of such composite mechanisms might lead to antidepressant and pro-cognitive (e.g. anti-inflammatory) (Wang & Li, 2024; Yin et al., 2024) or depressogenic and cognitive-impairing (e.g. lipid-lowering) (Rosoff et al., 2022; Taylor & Adhikari, 2025; van der Heijden & Houben, 2023) effects. There have also been historical (Zureik, Courbon, & Ducimetière, 1996) and enduring (Ye et al., 2023) concerns around suicidality and other adverse neuropsychiatric events (Pop et al., 2022) of statin treatment. There is therefore considerable interest in assessing their neuropsychiatric safety within at-risk populations and potential to repurpose statins for use in patients with depressive disorder (Chen et al., 2024; De Giorgi, Cowen, & Harmer, 2022; Jiang, Hu, McIntosh, & Shah, 2023).

Developing safe antidepressant treatments with innovative mechanisms of action or, conversely, identifying alternative physiopathological pathways leading to depression that can be targeted with new therapeutic agents is a research priority in mental health (Harmer, Duman, & Cowen, 2017). The potential for novel therapeutics, including repurposed compounds, can be assessed in early phase experimental medicine studies using validated measures based on the cognitive neuropsychological model of depression (Godlewska & Harmer, 2021). Depressive disorders are associated with negative biases in emotional processing (Roiser, Elliott, & Sahakian, 2012), and administration of conventional antidepressants can reduce these biases in healthy controls and depressed populations (Harmer et al., 2009). Reward learning appears similarly affected, as the presence of depressive symptoms can be associated with both reduced sensitivity to reward and heightened sensitivity to loss (Halahakoon et al., 2020), which normalize following antidepressant use (Herzallah et al., 2013; Scholl et al., 2017). Importantly, early changes in emotional biases or reward deficits may be associated with later antidepressant response (Harmer & Browning, 2022). Deficits in non-emotional cognition, such as memory and concentration, are also common in depression (Roiser & Sahakian, 2013); however, conventional antidepressants do not effectively treat these cognitive symptoms (Colwell et al., 2022; Prado, Watt, & Crowe, 2018).

People who are at-risk for developing depression due to the presence of predisposing factors, such as temperament patterns or adverse life events (Malhi & Mann, 2018), display affective biases that resemble those seen in patients with depressive disorders, and such biases also improve following treatment with traditional antidepressants (Di Simplicio, Norbury, & Harmer, 2012). In this context, social isolation and ensuing loneliness, which were exacerbated by the COVID-19 pandemic, have been associated with vulnerability to depression (Blanco, Wall, & Olfson, 2020), possibly via neurobiological mechanisms involving immune-metabolic functions (Pourriyahi, Yazdanpanah, Saghazadeh, & Rezaei, 2021). Anomalies in waking salivary cortisol (WSC), a stress hormone that suppresses immunological function, which can be used as a measure of hypothalamic–pituitary–adrenal (HPA) axis functionality, have been consistently linked to depression (Nandam, Brazel, Zhou, & Jhaveri, 2019) and correlate with emotional information biases in at-risk states (Le Masurier, Cowen, & Harmer, 2007). However, the combined influence of loneliness and HPA function on cognitive processes has not been formally examined.

Taken together, these observations offer an intriguing perspective to further define the neuropsychological profile associated with statin use. One of the mechanisms by which statins could express an antidepressant effect would be mediated by their activity on the HPA axis, whose correct functioning is tightly related to lipid metabolism, stress, inflammation, and mood regulation in animals and humans (Pellosmaa et al., 2015; Pellosmaa & Dougall, 2016; Pellosmaa & Dougall, 2018). These and other immune-metabolic processes, which may underlie the affective costs of social isolation and loneliness (Pourriyahi, Yazdanpanah, Saghazadeh, & Rezaei, 2021), appear influenced by statin administration. An observational study examining the neuropsychological effects of statins compared to several other drugs suggested that the former may be associated with less negative affective bias and reduced learning about reward loss (Gillespie et al., 2022). Findings of two further experimental medicine trials differed, though: seven days of the highly potent, moderately lipophilic atorvastatin worsened negative affective bias (De Giorgi et al., 2021), while seven days of the highly lipophilic, moderately potent simvastatin only mildly improved it (De Giorgi et al., 2022). Equally, a negative effect on these neuropsychological processes would constitute an important neuropsychiatric safety signal for statins; for example, previous studies of the medication rimonabant (Horder et al., 2009; Horder et al., 2012; Horder, Harmer, Cowen, & McCabe, 2010) led to withdrawal from the market after numerous reports of suicidal behavior (Sam, Salem, & Ghatei, 2011). Such discrepancies may be disentangled by investigating the neuropsychological effects of longer periods of statin treatment in individuals at risk for depression – such as those who have been exposed to high levels of social isolation and loneliness during the COVID-19 pandemic.

To test this hypothesis, we designed the current experimental medicine trial: the Oxford simvaSTatin and Emotional Processing (OxSTEP) study, which assesses the effects of 28-day simvastatin administration *versus* placebo on emotional processing, reward learning, working memory, and WSC in people who are at-risk for depression due to high levels of loneliness (Waters et al., 2023). In view of the evidence presented above, we hypothesized that longer-term simvastatin use in a group of predisposed participants may lead to positive changes on emotional processing and, secondarily, on reward learning and working memory, while also lowering WSC concentrations.

## Methods

### Ethics and study design

OxSTEP is an online, double-blind, parallel groups, randomized, gender-stratified, placebo-controlled, experimental medicine study approved by the University of Oxford Central University Research Ethics Committee (MS-IDREC R73946/RE001). The study protocol, which describes the methods in full, was prospectively registered on ClinicalTrials.gov (NCT04973800) and previously published (Waters et al., 2023). Members of the Oxford Health NIHR Biomedical Research Centre patient and public contributor pool with lived experience of depression were recruited during development of the study to consult remotely on the study population, design, and protocol. Further details of such discussions can be found in the study protocol.

This trial leveraged a novel approach to experimental medicine that involved a wholly online, remotely conducted design to resolve the recruitment and retention challenges due to the COVID-19 pandemic. This approach was also motivated by the lived experience consultation, which highlighted the increased accessibility and inclusivity that enrolling participants at a UK-nationwide level enables and the likely increase in sample diversity.

### Participants

Complete inclusion and exclusion criteria are in Supplementary Material, S1. Briefly, we aimed to recruit an overall healthy adult population (i.e. 21–65 year old, no medical or psychiatric illness, no regular medications) for whom it was safe to take the study medication and who were considered at-risk for developing depression due to a score ≥ 6 on the UCLA 3-item Loneliness Scale (Hughes, Waite, Hawkley, & Cacioppo, 2004), which indicates moderate to severe loneliness (i.e. a conservative score for the risk of depression (Matthews et al., 2022). The decision to select an at-risk group was guided by the lived experience consultation, which indicated this would (a) be more acceptable to participants in the context of long-term pharmacological intervention and (b) provide results of greater value and impact for patients.

The sample size calculation was based on the primary outcome of accuracy on a facial expression recognition task (FERT), a validated tool to assess emotional processing (Harmer et al., 2009) – see details in the Outcomes section. Using an online power calculator tool (https://www.sealedenvelope.com/power/continuous-superiority/), we computed that a sample size of 25 participants per study arm would give 0.9 power at a 0.05 significance level to detect changes of the magnitude of those seen in a previous key study using the emotional test battery (EMT) with antidepressant treatment [drug mean 10.64 *versus* placebo mean 5.96, standard deviation 5.1) (Harmer, Shelley, Cowen, & Goodwin, 2004)]. To account for potentially reduced sensitivity of online testing compared to face-to-face in a laboratory setting as well as lower predicted effect sizes of statins compared to conventional antidepressants, the calculated sample size was doubled to 50 participants per study arm. Withdrawn participants were replaced, and their data were not included in the analysis.

### Intervention/comparator

A researcher uninvolved in the study used an online randomization tool (https://www.sealedenvelope.com/simple-randomiser/v1/lists) to produce a randomization code in blocks of four, which was stored in a sealed envelope in a lockable cabinet. Enrolled participants were randomized to either simvastatin 20 mg, a safe dose used in previous clinical trials of simvastatin in depression (Abbasi et al., 2015; Gougol et al., 2015), or sucrose placebo, in identical capsules so that both participant and study researcher were masked to the study intervention/comparator. Randomized participants were posted 30 days of simvastatin/placebo to their address, including full written instructions of how and when to take them, and secure pre-paid postal boxes to return to the researchers with their saliva samples. Participants received daily automated text messages, reminding them to take the study capsule, and were required to have weekly contact with the study investigators to confirm adherence.

### Outcomes

All measures (i.e. WSC, questionnaires, and neuropsychological tasks) were taken both at baseline (pre-intervention) and after 28–30 days of study treatment (post-intervention). Waking saliva samples were collected by the participant and sent by post, then processed and stored as per standard operating procedure, and finally analyzed at the end of the study for WSC – methods are fully reported in Supplementary Material, S2. Questionnaires included: a bespoke COVID-19-related anxiety questionnaire (C19AQ), the Centre for Epidemiologic Studies-Depression scale (CES-D) (Radloff, 1977), an adapted social isolation scale (ELSA) (Bu, Abell, Zaninotto, & Fancourt, 2020), the Perceived Deficit Questionnaire (PDQ) (Strober et al., 2016), the Positive and Negative Affect Scale (PANAS) (Watson, Clark, & Tellegen, 1988), a bespoke side effects questionnaire (SEQ), the Snaith-Hamilton Pleasure Scale (SHAPS) (Snaith et al., 1995), and the State–Trait Anxiety Inventory (STAI) (Spielberger, Gorsuch, & Lushene, 1970). Feasibility outcomes for the remote design included participant dropout rate and adherence, successful completion of study tasks, and overall data quality and demonstration of expected main effect of condition across the tasks. Neuropsychological tasks involved the Oxford ETB (O-ETB) for emotional processing, including the FERT (with accuracy as the study primary outcome), the emotional categorization task (ECAT), and the emotional recall task (EREC) (Harmer et al., 2009), the probabilistic instrumental learning task (PILT) for reward learning (Pessiglione et al., 2006), the N-back task (NBT) for working memory (Redick & Lindsey, 2013). These tasks had been made suitable for remote online testing on a data protection-compliant platform and had also been previously validated in a similar study with observational design (Gillespie et al., 2022). A full description of the neuropsychological tasks is in Supplementary Material, S3.

### Study procedures

The study procedures are depicted in Supplementary Material, S4. Recruitment involved online adverts, whereby interested volunteers could access the Participant Information Sheet for the study. They were asked to complete a short pre-screening form, namely an online questionnaire referring to the inclusion/exclusion criteria (Supplementary Material, S1). Those who preliminarily met such criteria were offered a pre-screening call with a researcher, where key criteria were re-checked and informed consent was obtained via an online form.

Potential participants then had a video-call screening session with a study medic who collected information about physical and mental health history, including use of medications, using the Structured Clinical Interview for DSM-5 (SCID-5) (First, 2016) to confirm eligibility.

Enrolled participants were sent a secure parcel to their address, which included the study medications, the kits for collecting saliva samples, and instructions for all the above. They were also asked to contact the study researchers once their parcel was received. At the point of contact, a researcher called them to confirm the understanding of the study procedures and to schedule the research testing dates. Specifically, participants had to complete a remote baseline session, followed by 28–30 days of study medication, and then by a remote final day session. This structure was expressly designed to enable participants to arrange both sessions around their own commitments.

On an agreed initial day of research testing (pre-intervention), enrolled participants collected baseline waking cortisol saliva: one sample every 15 minutes to a total of four (Supplementary Material, S2). They then completed online questionnaires and neuropsychological tasks over about 1.5 hours, with a study researcher readily available over the phone to provide support as needed. On that evening, participants started taking the study medication, which they continued for 28–30 days (see Intervention/Comparator).

A second research testing session was scheduled after 28–30 days of study medications (post-intervention). On that morning, participants took their waking cortisol saliva samples again, then they repeated the questionnaires and a second version of the neuropsychological tasks. Although most studies previously employing these tests have used an entirely between-subject design (i.e. no pre-intervention *versus* post-intervention testing), these neuropsychological tasks have been shown to be robust to the effects of test–retest reliability (Thomas, Higgs, & Dourish, 2016). Therefore, outcomes were measured before and after study treatment to account for a more heterogenous cohort.

To measure the success of intervention/comparator masking, participants were asked to guess whether they were taking simvastatin or placebo.

### Statistical analysis

All data processing, analyses, and visualization were conducted using R (version 4.3.2, 2023), specifically using packages *lmer*, *rstatix*, *report*, and *brms.*

Participants’ socio-demographic characteristics and baseline loneliness scores (UCLA) were reported descriptively. WSC and repeated questionnaires (C19-AQ, CES-D, PANAS, SEQ, SHAPS, and STAI-state subscale) were analyzed via repeated measures analysis of variance (ANOVA) with group (simvastatin *versus* placebo) as the between-subject factor and time (pre-intervention *versus* post-intervention) as the within-subject factor. Feasibility outcomes for the remote design (participant dropout rate and adherence, successful completion of study tasks, and overall data quality and demonstration of expected main effect of condition across the tasks) were reported descriptively. For all neuropsychological tasks, data distributions were visually checked using boxplots, and data were excluded when variables were three standard deviations above or below the mean or when total accuracy was below 20%, as per previous studies (Gillespie et al., 2022). The resulting data were analyzed using repeated measures ANOVA with group as the between-subject factor and emotion/valence (O-ETB), win or loss (PILT), and trial condition (N-back) as the within-subject factors, and baseline values included as a covariate. Significant interactions were followed up using simple main effect analyses. When assumptions of equality of variances were not fulfilled, the Greenhouse–Geisser correction was used. Partial eta squared (η^2^) was reported for the main significant comparisons as a measure of effect size (η^2^ = 0.01, small effect; η^2^ = 0.06, medium effect; η^2^ = 0.14, large effect). Additionally, we conducted *post-hoc* Bayesian mixed-effects modelling for the FERT accuracy data (i.e. primary outcome) and computational reinforcement learning modelling for the PILT. Full details of the pre-specified and *post-hoc* statistical analyses are in Supplementary Material, S5.

## Results

**Figure 1.**
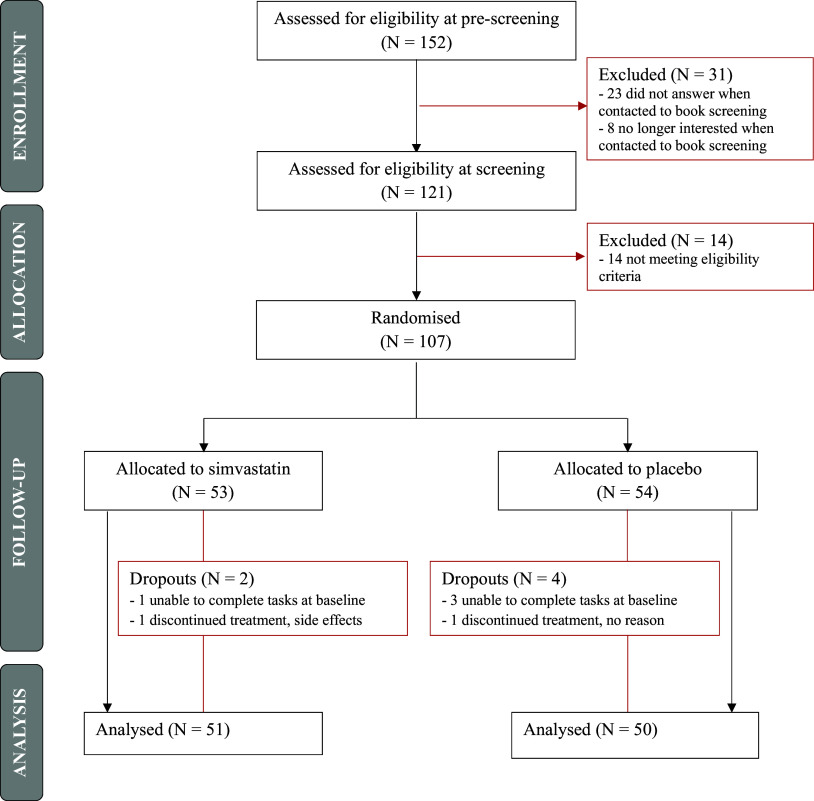
Study flow chart.

Participants enrolment started in July 2021 and was completed in February 2023 – study flow chart in Figure 1. Of the 152 people who were eligible at pre-screening, 121 proceeded to the screening visit. Of these, 14 were excluded as they did not meet eligibility criteria, and 107 participants were randomized. Participant dropout following randomization was 5.6% (N = 2 for simvastatin, N = 4 for placebo).

Participants’ socio-demographic characteristics and baseline loneliness scores are reported in Table 1: of the final study sample analyzed, 51 participants were randomized to simvastatin (33 females, mean age = 42.5 ± 13.6 years, mean BMI 24.8 ± 2.7) and 50 (33 females, mean age = 43.9 ± 12.5 years, mean BMI 24.4 ± 2.8) to placebo. More people randomly assigned to placebo had undergraduate or postgraduate education (84%) compared to those assigned to simvastatin (65%), and English was the first language for the majority (88%) of the study sample. Average loneliness scores at baseline were comparable between the two groups (mean UCLA 7.2 ± 1.2 for simvastatin, mean UCLA 7.4 ± 1.2 for placebo).

**Table 1.** Participants’ socio-demographic characteristics and baseline loneliness scores

		Simvastatin *N = 51*	Placebo *N = 50*
Sex assigned at birth		33 F/18 M	33 F/17 M
Age		42.5 (13.6)	43.9 (12.5)
English as first language		44 (86.3%)	45 (90.0%)
Education	High school/college	18 (35.3%)	8 (16.0%)
	Undergraduate	20 (39.2%)	25 (50.0%)
	Postgraduate	13 (25.5%)	17 (34.0%)
Tobacco/day		2.0 (1.0)	1.6 (1.1)
Alcohol units/week		5.8 (3.2)	5.4 (3.1)
BMI		24.8 (2.7)	24.4 (2.8)
Family history of mental disorder		25 (49.0%)	23 (46.0%)
UCLA		7.2 (1.2)	7.4 (1.2)

*Note:* Values are means (standard deviations) but for categorical variables.

Abbreviations: BMI, body mass index; UCLA, loneliness score.

Participants’ baseline (pre-intervention) and final (post-intervention) measures for WSC (averaged across the four samples) and all questionnaires are reported in Table 2: notably, average scores on the CES-D at baseline were above the threshold of 16 for ‘at-risk for clinical depression’ at baseline (17.84 ± 9.57 for simvastatin, 19.12 ± 10.72 for placebo) but not on the final visit (14.33 ± 9.13 for simvastatin, 15.88 ± 11.63 for placebo); however, there was no significant group-time interaction for any of the questionnaires administered or for cortisol (F’s < 1.82, p’s > 0.20). Adequate intervention/comparator masking was achieved (χ^2^ = 0, df = 1, p = 1.000). Missing data rates (including data excluded for being incomplete, invalid, or an outlier) were minimal (<5% for all but one task - an early version of the N-back task instructions provided to participants included a mistake, sensitivity analysis was used on these participants, N = 10) and comparable to typical in-person experimental studies – details in Supplementary Material, S6. Supplementary figures for main outcomes (i.e. WSC, CES-D, and neuropsychological tasks) are in Supplementary Material, S7.

**Table 2.** Participants’ baseline (pre-intervention) and final (post-intervention) measures

		Simvastatin *N = 51*		Placebo *N = 50*			
		Baseline	Final	Baseline	Final	*F*	*p*
WSC μg/dL		0.35 (0.12)	0.33 (0.13)	0.33 (0.14)	0.32 (0.15)	0.41	0.524
C19AQ		25.00 (5.14)	24.80 (5.56)	26.04 (6.57)	25.92 (6.45)	1.82	0.180
CES-D		17.84 (9.57)	14.33 (9.13)	19.12 (10.72)	15.88 (11.63)	0.71	0.402
ELSA		38.31 (9.24)	40.59 (8.41)	38.82 (9.62)	39.98 (9.77)	0.21	0.651
PDQ		13.69 (4.08)	12.73 (4.00)	13.62 (3.86)	12.78 (3.30)	<0.01	0.927
PANAS	Positive	29.43 (7.97)	30.76 (8.62)	29.56 (8.76)	31.20 (9.24)	0.10	0.752
	Negative	18.80 (6.37)	16.22 (6.43)	19.16 (6.83)	17.10 (7.12)	0.54	0.466
SEQ		2.06 (2.54)	3.60 (3.65)	2.10 (2.68)	3.28 (3.45)	1.00	0.370
SHAPS		2.08 (2.86)	1.31 (2.23)	2.14 (2.73)	1.44 (2.87)	0.07	0.796
STAI	Trait	45.20 (11.71)	–	46.26 (12.24)	–	–	–
	State	43.57 (5.47)	41.49 (10.62)	43.40 (5.77)	44.84 (12.13)	2.16	0.144

*Note:* Values are means (standard deviations); F’s indicate between-group interactions on the final day (post-intervention), where baseline scores were used as within-subject factors.

Abbreviations: WSC, waking salivary cortisol; C19AQ, COVID-19-related anxiety questionnaire; CES-D, Centre for Epidemiologic Studies-Depression scale; ELSA, adapted social isolation scale; F, F-statistics; p, p-value; PDQ, perceived deficit questionnaire; PANAS, positive and negative affect scale; SEQ, side effects questionnaire; SHAPS, Snaith-Hamilton Pleasure Scale; STAI, State–Trait anxiety inventory.

A summary of findings for the neuropsychological tasks’ outcomes is in Table 3.

**Table 3.** Summary of findings for neuropsychological tasks

Neuropsychological task	Outcome	*F*	*p*	*η*^*2*^
FERT	Accuracy	F_6_ = 0.55	0.773	5.68e−03
	Unbiased hit rate	F_6_ = 0.67	0.678	6.95e−03
	Misclassification (a)	F_6_ = 1.14	0.340	0.01
	Misclassification (b)	F_6_ = 0.64	0.699	6.64e−03
	Reaction time	F_6_ = 0.52	0.791	5.53e−03
ECAT	Accuracy	F_1_ = 4.34e–03	0.948	4.65e−05
	Response time	F_1_ = 1.34	0.251	0.01
EREC	Correct words	F_1_ = 0.05	0.832	4.97e−04
	Incorrect words	F_1_ = 1.08	0.301	0.01
PILT	Win vs loss	F_1_ = 1.36	0.246	0.01
	Win vs loss of last 20 trials	F_1_ = 1.95	0.166	0.02
	Response time of win vs loss	F_1_ = 2.41	0.124	0.02
N-back	Accuracy	F_3_ = 0.21	0.889	2.71e−03
	Hits	F_3_ = 0.90	0.444	0.01
	False alarms	F_3_ = 0.60	0.614	7.54e−03
	Response time	F_3_ = 0.58	0.629	7.74e−03

*Note:* F’s indicate between-group interactions on the final day (post-intervention) with condition as a within-subject factor and baseline scores included as a covariate.

Abbreviations: ECAT, emotional categorization task; EREC, emotional recall task; η^2^, partial Eta squared; F, F-statistics; FERT, facial expression recognition task; Misclassification (a), misclassify this emotion; Misclassification (b), misclassify this emotion as a different emotion; p, p-value; PILT, probabilistic instrumental learning task.

### Oxford emotional test battery (O-ETB, emotional processing)

*FERT*: As expected, for the primary outcome of percentage accuracy of emotion recognition, the task produced a significant main effect of emotion (F_6_ = 16.61, p < 0.001; η^2^ < 0.14). Accuracy was highest for happy faces and lowest for fearful faces, comparable to other similar studies completed in the laboratory (De Giorgi et al., 2021; De Giorgi et al., 2022).

**Figure 2.**
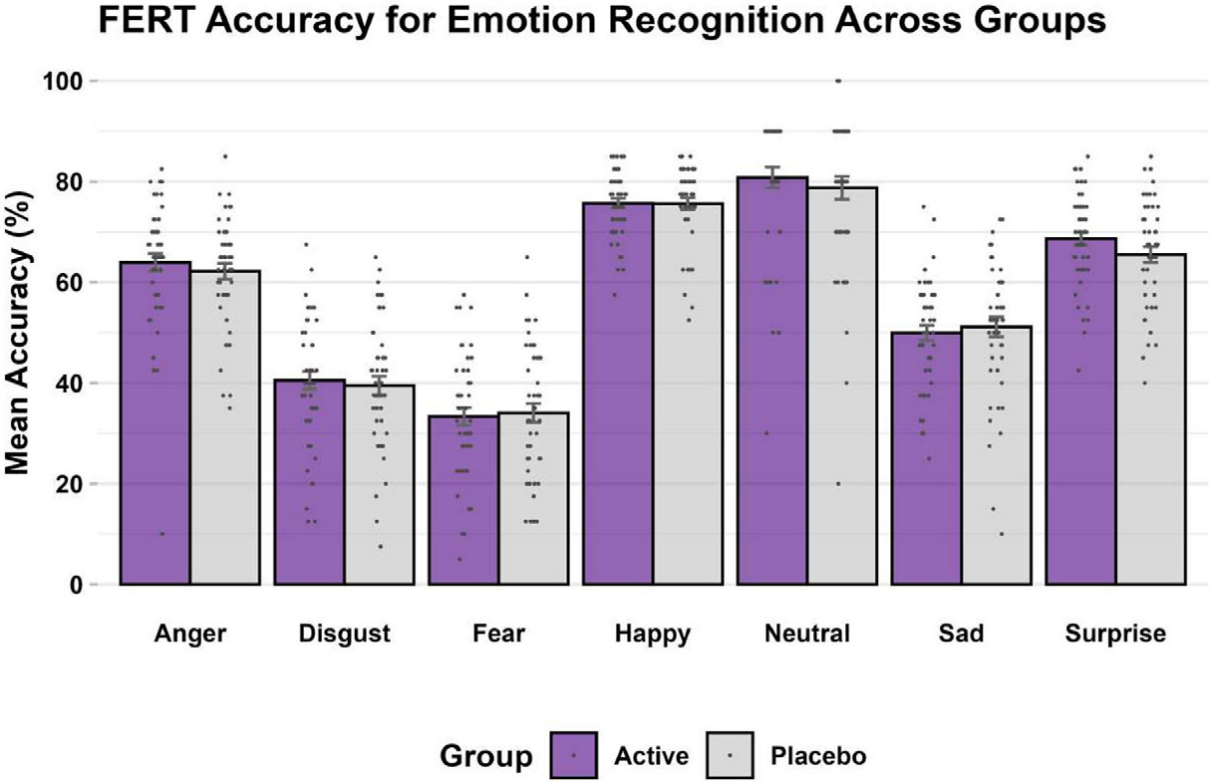
FERT, accuracy (primary outcome) at endpoint. *Note:* Bars are mean accuracy scores expressed as percentages (purple: simvastatin, gray: placebo), error bars correspond to standard errors, and dots correspond to individual subject values.

The interaction between emotion and group was not significant and very small (F_6_ = 0.55, p = 0.77; η^2^ < 0.01) – see Figure 2. Moreover, there was no significant group-emotion interaction for any of the other outcome measures (i.e. unbiased hit rate, misclassifications, and reaction time) for this task (F_6_’s < 1.15, p’s > 0.20, η^2^’s < 0.01). This remained the same whether controlling for baseline values as a covariate or not.

Briefly, findings for the *post-hoc* Bayesian mixed-effects modelling were consistent with those of frequentist-based modelling (i.e. repeated measure ANOVA) for the FERT accuracy data. Bayes Factor (BF) analyses showed strong evidence for a lack of interaction between group and emotion on unbiased hit rate (BF_10_ = 0.003). Full details are in Supplementary Material, S8.

*ECAT, EREC*: As expected, the task produced a significant main effect of valence on EREC intrusions, with participants recalling more positive intrusions than negative intrusions (F1 = 35.33, p < 0.001; η2 = 0.25), in keeping with other studies performed in the laboratory (De Giorgi et al., 2021; De Giorgi, Quinton et al., 2022).

There were no significant group-valence interactions for any of the ECAT (i.e. accuracy and response time) and EREC (i.e. correct words and incorrect words) outcomes measured (F_1_’s < 1.35, p’s > 0.20, η^2^’s < 0.01).

### Probabilistic instrumental learning task (PILT, reward learning)

As expected, participants learned the association between the stimuli for reward and loss across the trials, with participants selecting the stimuli with a high probability of reward 74.8% of the time and selecting the stimuli with a high probability of loss 29.54% of the time across the last 20 trials of each block (De Giorgi et al., 2021; De Giorgi, Quinton et al., 2022).

There was no significant effect of simvastatin (F_2_’s < 2.41, p’s > 0.20, η^2^’s < 0.01) on any of the outcome measures (i.e. win *versus* loss, win *versus* loss last 20 trials, win *versus* loss response time). *Post-hoc* computational reinforcement learning modeling did not materially change these results, as shown in Supplementary Material, S8.

### N-back task (NBT, working memory)

As expected, there was a significant main effect of condition, with working memory performance reducing as task demands increased (F_3_ = 116.03, p < 0.001; η^2^ = 0.59) (Gillespie et al., 2022). However, no significant differences between the simvastatin and placebo groups were identified (F_3_’s < 0.90, p’s > 0.20, η^2^’s < 0.01) for any of the outcomes measured (i.e. accuracy, hits, false alarms, and response time).

## Discussion

In this study of a sample of people at risk for depression due to high levels of loneliness, we investigated the effects of 28-day simvastatin on a battery of neuropsychological tests, questionnaires, and WSC, using an innovative remote design for conducting experimental medicine trials. This design was approved by the University of Oxford Central University Research Ethics Committee (MS-IDREC R73946/RE001) as an experimental medicine study in the United Kingdom; the safety of the participants was ensured via extensive medical and psychiatric screening and availability of a medic on-call as required. The study completion rate, overall task adherence and data quality, and evidence of anticipated main effects of task condition were comparable to those seen for in-person laboratory studies (De Giorgi et al., 2021; De Giorgi, Quinton et al., 2022). This suggests the remote design was successful and comparable to in-person administration. In a large sample (101 participants), there was no significant effect of simvastatin administration compared with placebo on measures of emotional processing, reward learning, and working memory, nor on any of the questionnaires and WSC levels. In particular, accuracy in FERT, the study’s primary outcome, was not affected by simvastatin use, and this null result was confirmed by *post-hoc* Bayesian analysis. Similarly, other cognitive tasks assessing ECAT, EREC, PILT, and working memory capacity (N-back) did not reveal significant differences between the two groups. WSC response and self-reported measures of mood/affect, anxiety, and other psychosocial functions (C19AQ, CES-D, ELSA, PDQ, PANAS, SHAPS, and STAI-s) also did not show significant group-time interactions, suggesting that simvastatin does not alter waking cortisol or meaningfully alter mood. Taken together, these findings indicate that 28-day administration of simvastatin does not have the hypothesized positive effects on emotional and other cognitive processes, nor on cortisol regulation, in individuals likely at risk for depression.

The absence of significant effects observed in OxSTEP contrasts with prior studies that suggested statins might affect negative emotional biases or influence reward learning (De Giorgi et al., 2021; De Giorgi, Quinton et al., 2022; Gillespie et al., 2022). Previous experimental medicine trials had shown mixed results regarding the effects of statins on mood and cognition, with shorter statin regimens indicating modest changes in emotional processing (De Giorgi et al., 2021; De Giorgi, Quinton et al., 2022). The rationale for this study indeed stemmed from the assumption that a longer administration period of 28 days would be more effective, especially in individuals vulnerable to depression due to loneliness. Our findings, however, align with more recent literature (De Giorgi et al., 2023; De Giorgi, Cowen, & Harmer, 2022; De Giorgi et al., 2022; Hang et al., 2021; Lee et al., 2021; Zhang et al., 2022), and especially with the largest, most robust clinical trial to date (Husain et al., 2023), which questions any direct antidepressant potential of statins. We used a relatively low dosage of 20 mg simvastatin, which is in line with previous trials investigating this medication in depression (Abbasi et al., 2015; Gougol et al., 2015; Husain et al., 2023), but it is possible that higher doses are needed to see an effect on behavioral tasks. The immune-metabolic model of depression suggests that factors like inflammation and lipid metabolism may play a significant role in mood regulation, but this trial does not support the notion that statins can independently ameliorate depressive symptoms or cognitive biases in at-risk populations with short-term treatment. Still, it remains possible that any presumed antidepressant activity of statins in clinical practice, as proposed by other studies (De Giorgi et al., 2021; Molero et al., 2020; Xiao et al., 2023; Yang et al., 2024), might relate to neural pathways that are not accounted for by the neuropsychological tasks employed in OxSTEP or that would require exploration via more sensitive measures on functional magnetic resonance imaging (fMRI) (Godlewska & Harmer, 2021). Further, the benefit of statins may only be evident in older populations (Gillespie et al., 2022; Redlich et al., 2014), compared to our relatively young sample, who are the most likely to be prescribed these drugs for cardiovascular and metabolic ailments in an observational setting and who might present with age-related higher baseline inflammation (Ferrucci & Fabbri, 2018).

Conversely, the lack of negative effects on any of the assessed neuropsychological domains or questionnaires for mood/anxiety provides reassurance against previously described adverse effects of statins on emotional and cognitive processes and disorders (Leutner et al., 2021; Rosoff et al., 2022; Taylor & Adhikari, 2025; van der Heijden & Houben, 2023), including suicidality (Zureik, Courbon, & Ducimetière, 1996) – as it had been the case instead with rimonabant (Horder et al., 2009; Horder et al., 2012; Horder, Harmer, Cowen, & McCabe, 2010). Again, these results are in line with more recent evidence advocating the safety of statins in people with mental illness (De Giorgi et al., 2024; Ye et al., 2023), thus supporting ongoing prescribing of these medications in this population at high risk for cardiovascular morbidity and mortality (Rajan et al., 2020).

### Limitations

Several limitations may have contributed to the null findings of the OxSTEP study.

First, the remote study design may lead to possible issues in ensuring consistent participant engagement and precise measurement of outcomes in neuropsychological tasks. At-home testing introduces a less rigorously controlled environment and may result in different emotional states that introduce potential biases when compared with in-person testing. Nevertheless, this novel approach had been previously validated in an observational study (Gillespie et al., 2022), and the current study had the additional benefit of participants being supported by a study researcher via phone during the testing sessions. All data quality checks indicated anticipated effects of task condition and minimal data loss due to poor performance. Moreover, this setup allowed us to reach a UK-wide, more geographically diverse population, hence potentially producing more generalizable results as suggested for this kind of study (Clark et al., 2019). Other advantages reported by our patient and public involvement and engagement group included avoidance of unnecessary travel by study participants, which saved time and money and further reduced barriers to participation .

Another particular challenge with remote studies, which is partly shared by in-person studies, is ascertaining whether participants have taken the medications as prescribed. There may be advantages in using independent measures of concordance, for example, salivary drug concentration or similar biomarkers. Indeed, lipophilic statins such as simvastatin have been reported to increase plasma cortisol levels (Sahebkar, Rathouska, Simental-Mendía, & Nachtigal, 2016), but no such effect was observed in the WSC measures obtained in this study. Although all participants self-reported a strict compliance with daily medication intake and this was aided by daily text reminders, we did not have any other objective, independent measure of compliance.

Second, as study recruitment occurred between July 2021 and February 2023 across several unpredictable waves of COVID-19 lockdowns and subsequent reopening, we could not account for the effects that these may have had on our findings, while the remote design allowed for this study to be resilient to changes in government policies as well as participants’ infections. We did not serially record participants loneliness scores but rather utilized this as a screening questionnaire, as those meeting the cut-off were presumed ‘at-risk’ due to loneliness. Due to the subjective nature of loneliness, unpredictability of lockdown measures or potential shielding at the time, we cannot presume that this risk remained stable across the 28 days.

Third, the sample size of 101 participants, despite being well-powered for the primary outcome and significantly larger than earlier studies (De Giorgi et al., 2021; De Giorgi, Quinton et al., 2022), may have lacked the statistical power to detect more subtle cognitive or emotional effects of simvastatin, especially in a healthy population at risk for depression but without active symptoms. Furthermore, the inclusion criteria targeting loneliness as a risk factor for depression do not fully capture the heterogeneity of depression vulnerability (Malhi & Mann, 2018). Indeed, loneliness is a complex construct, and its relationship with emotional processing might require longer periods of intervention than the 28-day simvastatin administration.

Lastly, the study measured WSC as an indicator of HPA axis function, but other markers of immune-metabolic response were not assessed due to the constraints of the remote study design. Since statins exert anti-inflammatory effects that might influence mood via pathways beyond cortisol regulation (Pellosmaa et al., 2015; Pellosmaa & Dougall, 2018), the absence of such measures could have limited the understanding of the broader biological impacts of simvastatin. Additionally, statins would also affect lipid metabolism with possible consequences on brain functioning (Pellosmaa et al., 2015; Pellosmaa & Dougall, 2018), but this was not assessed in this study.

## Conclusions

In conclusion, 28-day administration of simvastatin did not lead to changes in emotional processing, reward learning, working memory, or WSC levels in individuals likely at-risk for depression due to loneliness. These findings contrast with earlier studies that suggested potential mood benefits of statins but are consistent with more recent literature refuting their independent efficacy in the treatment or prevention of depressive episodes. The lack of adverse neuropsychological mechanisms adds to the evidence base supporting the safety, but not efficacy, of statins in people with mental disorders. A definitive answer regarding any potential antidepressant effect of statins may come from further research testing these medications in people with a clinical diagnosis of depression and raised markers of immune-metabolic dysfunction (Otte et al., 2020). Meanwhile, suboptimal prescribing of statins in people with depressive disorders due to concerns around adverse neuropsychiatric effects (Ljung, Köster, Björkenstam, & Salmi, 2021; Magin et al., 2021; Park, Tickle, & Cutler, 2022; Ter Braake et al., 2024; Vinuesa-Hernando et al., 2024) should be avoided.

## Supporting information

De Giorgi et al. supplementary material De Giorgi et al. supplementary material

## Data Availability

The data produced by this study will be available from the corresponding author upon reasonable request.

## Supplementary material

The supplementary material for this article can be found at http://doi.org/10.1017/S0033291725001187.
