# Supplementary material for: Effects of 28-day simvastatin administration on emotional processing, reward learning, working memory, and salivary cortisol in healthy participants at-risk for depression: OxSTEP, an online experimental medicine trial
Source: Psychol Med. 2025 May 22;55:e155. doi: 10.1017/S0033291725001187 (PMC12115272; doi:10.1017/S0033291725001187)
Supplement: De Giorgi et al. supplementary material [file S0033291725001187sup001.docx]

**Effects of 28-day simvastatin administration on emotional processing, reward learning, working memory, and salivary cortisol in healthy participants at-risk for depression: OxSTEP, an online experimental medicine trial**

SUPPLEMENTARY MATERIAL

***Supplementary Material, S1.*** Inclusion and exclusion criteria

| Inclusion Criteria |
| --- |
| Male or female |
| Aged 21-65 years |
| At-risk for depression as measured by a score $\boldsymbol{\geq}$6 on the UCLA 3-item Loneliness Scale |
| Body Mass Index (BMI) in the range of 18-30 |
| Willing and able to give informed consent for participation in the study |
| Registered with a GP and consenting to GP being informed of participation in the study |
| Currently living in the UK and sufficiently fluent English to understand and complete the tasks |
| Able to access and use a computer with internet |
| Able to complete online questionnaires and tasks |
| \| Exclusion Criteria \| \| --- \| |
| Currently on any regular prescribed medications (except the contraceptive pill), unless unlikely to compromise safety or affect data quality in the opinion of the Investigator |
| History or current significant psychiatric illness (other than past [>6 months] episodes of depression or anxiety) |
| Current alcohol or substance misuse disorder (<6 months) |
| History of, or current significant hepatic disease |
| History of, or current significant neurological condition (e.g. epilepsy) |
| History of haemorrhagic stroke or deep brain structures stroke |
| Known hyperglycaemia/pre-diabetes/diabetes |
| Known hypersensitivity to the study drug (i.e. simvastatin) or sucrose |
| Pregnant, breast feeding, or women of child-bearing potential not using appropriate contraceptive measures |
| Participation in a study that uses the same or similar computer tasks (apart from the N-back) as those used in the present study |
| Participation in a study that involves the use of a medication within the last 3 months |
|  |

***Supplementary Material, S2.*** Waking Salivary Cortisol methods

On their first day, participants will take waking cortisol saliva samples – four samples taken 15 minutes apart as per standard operating procedure. Each sample is collected with a cotton swab and salivette(1), pre-labelled with the anonymised subject ID, and marked 1 to 4. Participants are instructed to take the samples according to this order, remaining in bed with the lights off and without food or drink. They are asked to mark the time of collection on each tube, before returning them by post in the secure pre-paid box. On the morning of the final day session, participants will take another waking saliva sample. Saliva samples, which arrive by post, will be processed and stored as per standard operating procedure by a trained researcher. Samples will be centrifuged at 1000g for 2 minutes before being transferred into labelled 5mL polypropylene tubes under a Class II biohazard hood. Tubes will be placed in a storage box within a clinical freezer at -20 degrees C. This has been tested and validated to be a reliable method of cortisol measurement(2-4).

***Supplementary Material, S3.*** Neuropsychological tasks

*Oxford emotional test battery (O-ETB)*

The Oxford emotional test battery (O-ETB) (5) assesses emotional processing and includes the facial expression recognition task (FERT), the emotional categorisation task (ECAT), and the emotional recall task (EREC).

In the FERT, facial expressions of six emotions (anger, disgust, fear, happy, sad, and surprise) and a neutral expression are randomly displayed on the screen for 500ms. Participants must respond by identifying the expression as quickly and accurately as possible. Facial expressions are adapted from the Karolinska directed emotional faces set(6) and are depicted at a range of intensity levels(7). The primary outcome is accuracy at identifying the correct emotion. Additional outcomes are misclassifications and mean reaction times. To assess discriminability (d’, a measure of sensitivity), and response bias (β, a measure of conservativeness)(8), a signal detection analysis is carried out. Unbiased hit rate is used to measure accuracy whilst considering response bias.

In the ECAT, twenty positively- and twenty negatively-valenced personality characteristic words(9) are randomly displayed on the computer screen for 500ms. Participants must indicate, as quickly and accurately as possible, whether they would ‘like’ or ‘dislike’ to be described by this word. Accuracy, as well as mean reaction time, discriminability, and response bias are assessed. After a delay of around 10-20 minutes, participants carry out the EREC, a free-recall task. Here, participants are given four minutes to type the personality characteristics words they remember from the ECAT. The number of correctly- and falsely-recalled positive and negative words are assessed.

*Probabilistic instrumental learning task (PILT)*

The probabilistic instrumental learning task (PILT) assesses reward learning (adapted from Pessiglione, Seymour (10)). In this task, the aim is to win as much money as possible by picking between two symbols which are displayed on the computer screen for 4,000ms. Participants begin with £1. Across three blocks, there are 90 win trials where one symbol of each pair will result in winning £0.20 and the other in no change; and 90 loss trials where one symbol of each pair will result in no change and the other in losing £0.20. For both win and loss trials, one symbol will result in the better outcome 70% of the time, and the other 30% of the time. Feedback on the outcome is given after each trial. Participants have to use this to learn over time which symbols are associated with high-probability to win, and which are associated with high-probability to lose. Probability of choosing the winning symbol in win trials and the losing symbol in loss trials are assessed, as well as end total money and amount won and lost.

*N-back*

The N-back task assesses working memory (11). Participants respond to whether letters, appearing sequentially on the screen, match the letter presented N-trials before. Four conditions will be used: 0-back, where participants respond by pressing the ‘m’ or ‘n’ key (yes or no, respectively), if the letter presented is an ‘X’ or not. Followed by 1-back, 2-back, and 3-back conditions, asking whether the letter is the same as it was one, two and three trials ago, respectively. This task employs a block design, each condition has 2 blocks of 20 trials. Accuracy and mean response times for correct trials for each condition are assessed, as well as discriminability and response bias.

***Supplementary Material, S4.*** Study procedures


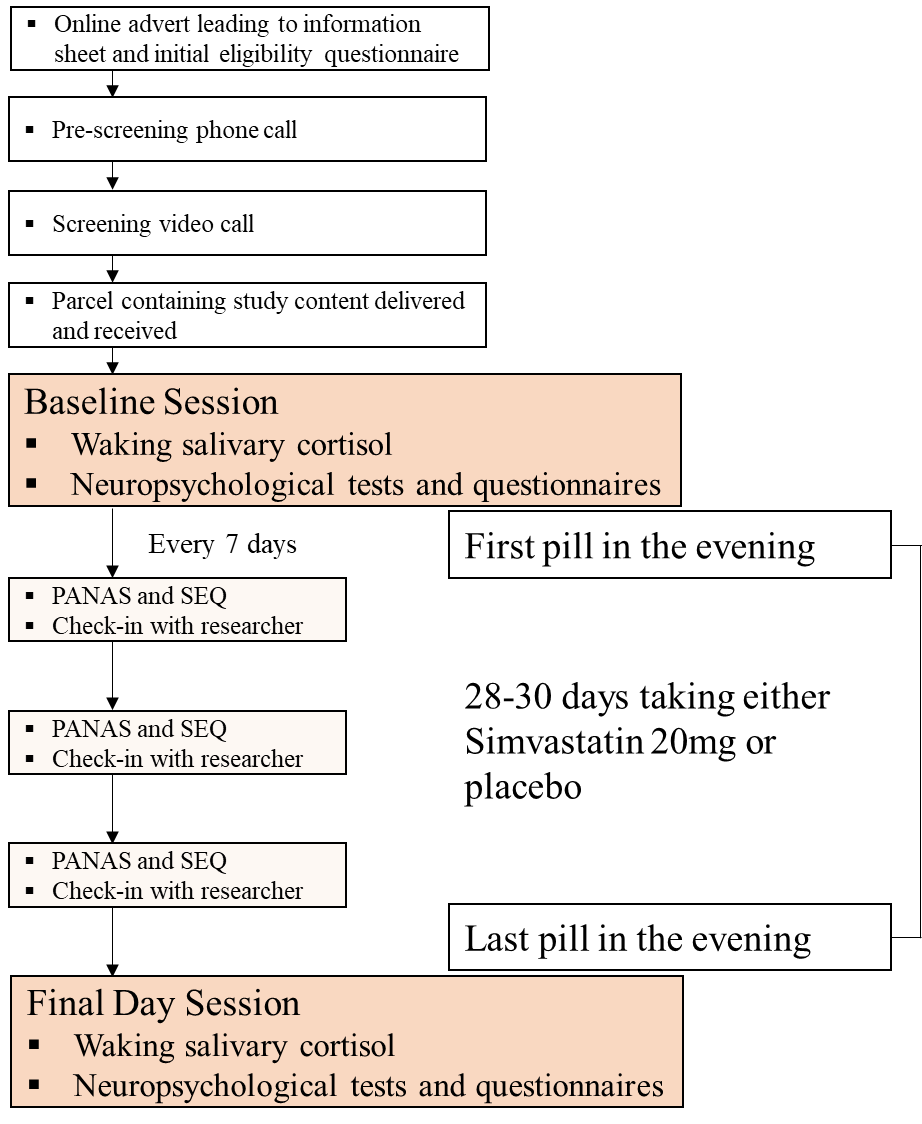


PANAS: Positive and Negative Affect Schedule; SEQ: Side-Effects Questionnaire

***Supplementary Material, S5.*** Statistical plan

Demographic and baseline measures will be reported descriptively. Loneliness, mood, anxiety, side-effects, and salivary cortisol measures will be analysed using repeated measures analysis of variance (ANOVA) with group (simvastatin *versus* placebo) as the between-subject factor and time (baseline *versus* final day session) as the within-subject factor. Feasibility outcomes for the remote design will be reported such as dropout rate, including within study exclusions, adherence, and missing data rates.

Data distributions will be visually checked for all neuropsychological tasks, using boxplots. Extreme outliers (i.e., data values lying more than three times outside the interquartile range) will be excluded. The resulting data will be analysed using ANOVA, with group as the between-subject factor and time as a within-subjects factor. The individual tasks will be analysed with the following additional within-subjects factors: for FERT, emotion; for ECAT and EREC, valence; for PILT, win or loss; and for N-back, trial condition.

Any significant interactions will be followed up using simple main effect analyses. When assumptions of equality of variances are not fulfilled, the Greenhouse-Geisser procedure will be used to correct the degrees of freedom.

*Post-hoc analyses*

*Post-hoc* Bayesian mixed-effects modelling was undertaken using R Software (version 2024.12.0) with the *brms* package (Bürkner, P.-C. Advanced Bayesian multilevel modeling with the R package brms, arXiv preprint arXiv:1705.11123) for the FERT accuracy data (i.e., primary outcome). The prior distribution was set to Gaussian, and participant was included as a random effect. The modelling structure was selected to match the a priori frequentist analyses. The model was expressed as: outcome [follow-up] ~ group * emotion + outcome [baseline] + (1 | participant), where the factor for emotional face expressions (i.e., emotion) was coded with neutral facial expressions as the reference category. Effects were considered significant if the 95% credible intervals did not contain zero. Follow-up Bayes Factor tests were conducted to assess the strength of evidence for or against the alternative hypothesis.

For the PILT, we fitted observed behavioural data to computational reinforcement learning models using a previously validated modelling approach for this task (12, 13). In accordance with the non-model analysis, model parameters were combined for both trial types in combination, as done in previous work (14). The model consisted of two key parameters: learning rate (describing the rate by which learning changes beliefs) and outcome sensitivity (describing) the effective magnitude associated with outcomes as they are experienced. The parameter recovery and modelling selection process have been previously described (12, 13).

***Supplementary Material, S6.*** Missing data rates

|  | **FERT** | **ECAT** | **EREC** | **PILT** | **N-Back** | **Questionnaires** | **Cortisol** | | |
| --- | --- | --- | --- | --- | --- | --- | --- | --- | --- |
| ***Data Incomplete*** | 1 | 0 | 0 | 1 | 0 | 0 | 0 | | |
| ***Data Invalid*** | 1 | 1 | 2 | 2 | 0 | 0 | 1 | | |
| ***Outliers*** | 1 | 4 | 1 | 1 | 19 | 0 | 4 | | |
| Table shows numbers of missing data rates for each task out of N = 101. Examples of when data has been deemed invalid has been when wrong versions of the tasks have been used (n = 1), task instructions have been misunderstood (n = 1), and participant computers have crashed during critical periods during a task (n = 2). Data was excluded when variables were 3 standard deviations above or below the mean or when total accuracy was below 20%. An early version of the N-back task instructions provided to participants included a mistake, sensitivity analysis was used on these participants (n = 10). | | | | | | | |  |  |

***Supplementary Material, S7.*** *Supplementary figures.* Bars are mean (purple: simvastatin, grey: placebo), error bars correspond to standard errors, dots correspond to individual subject values


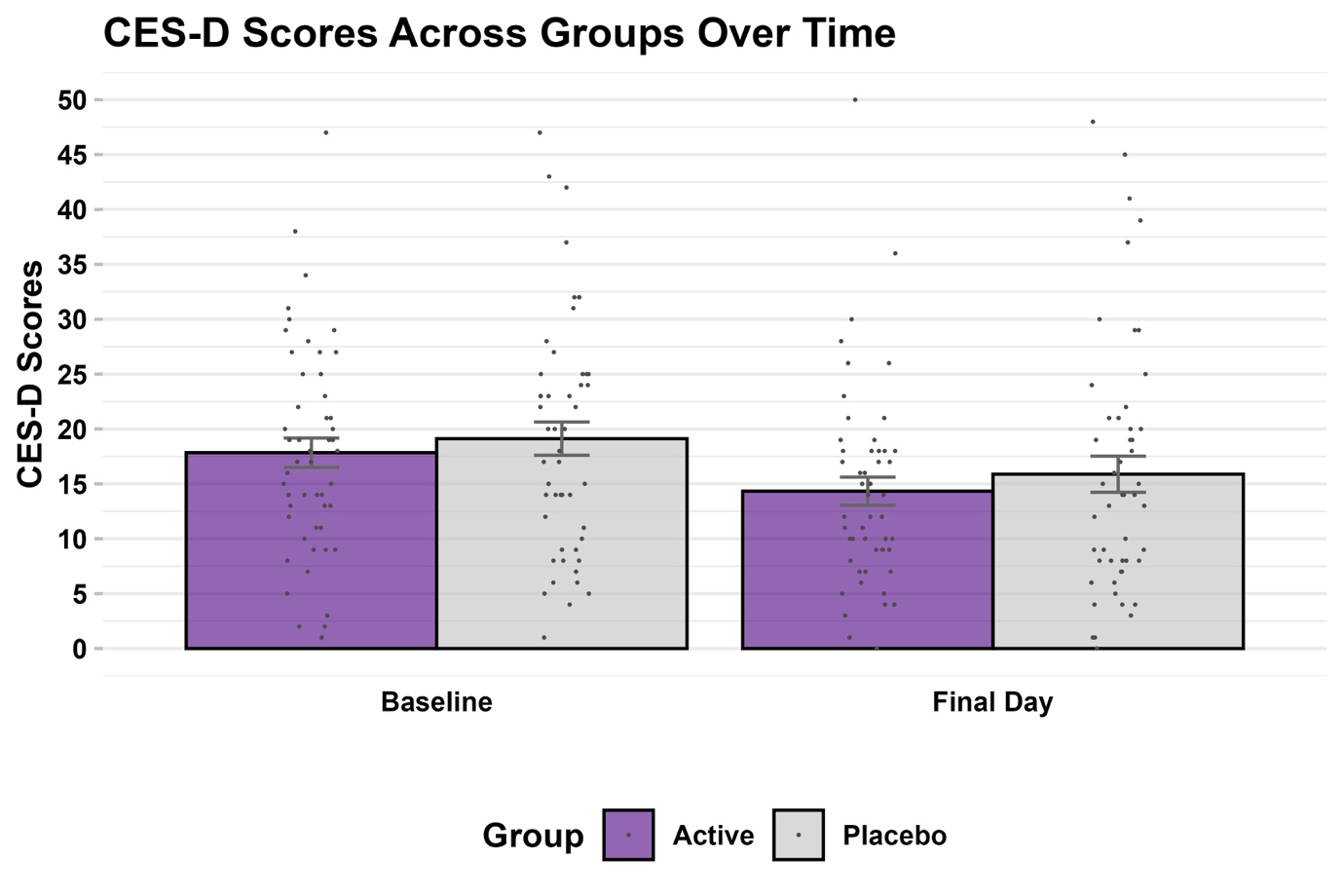

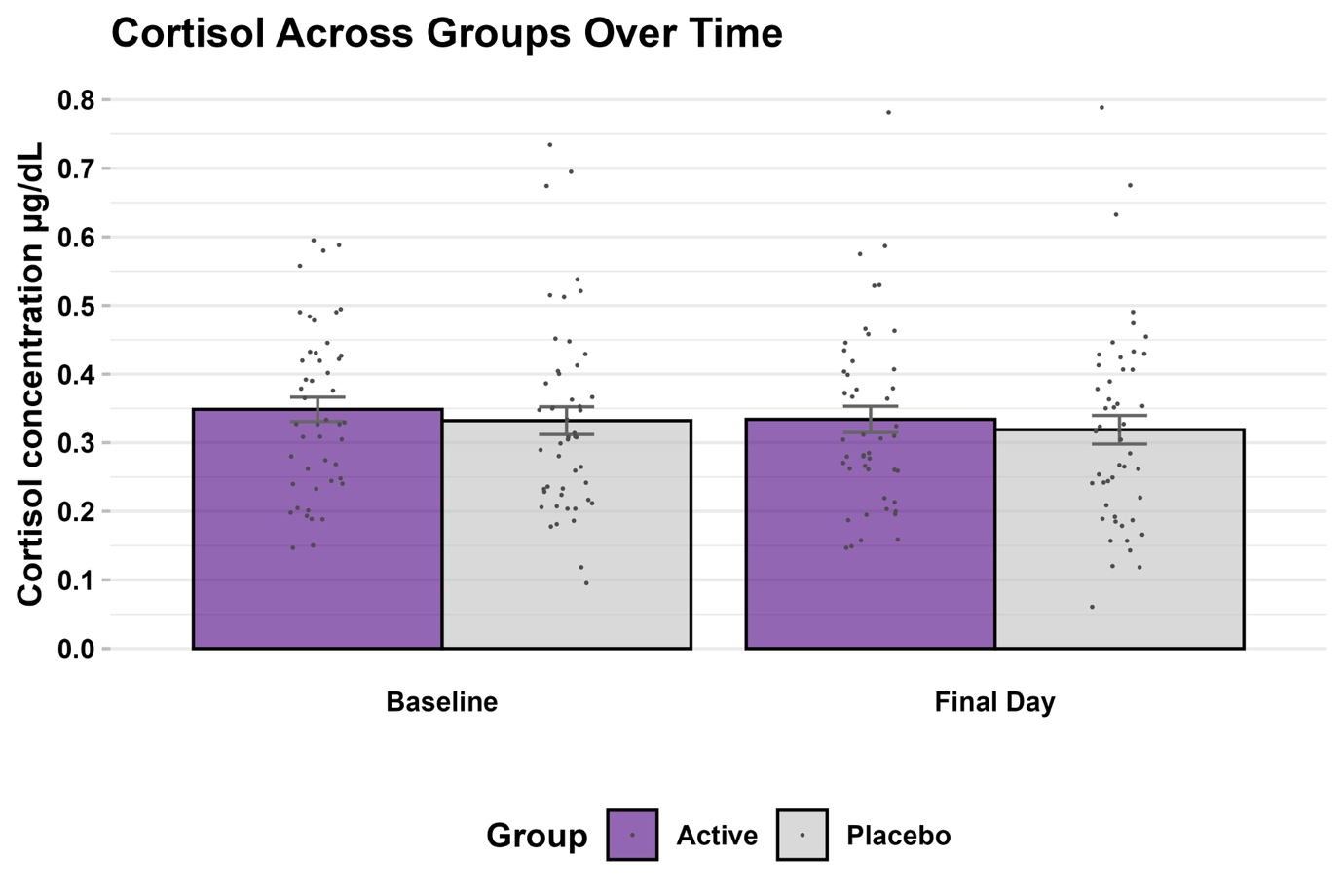

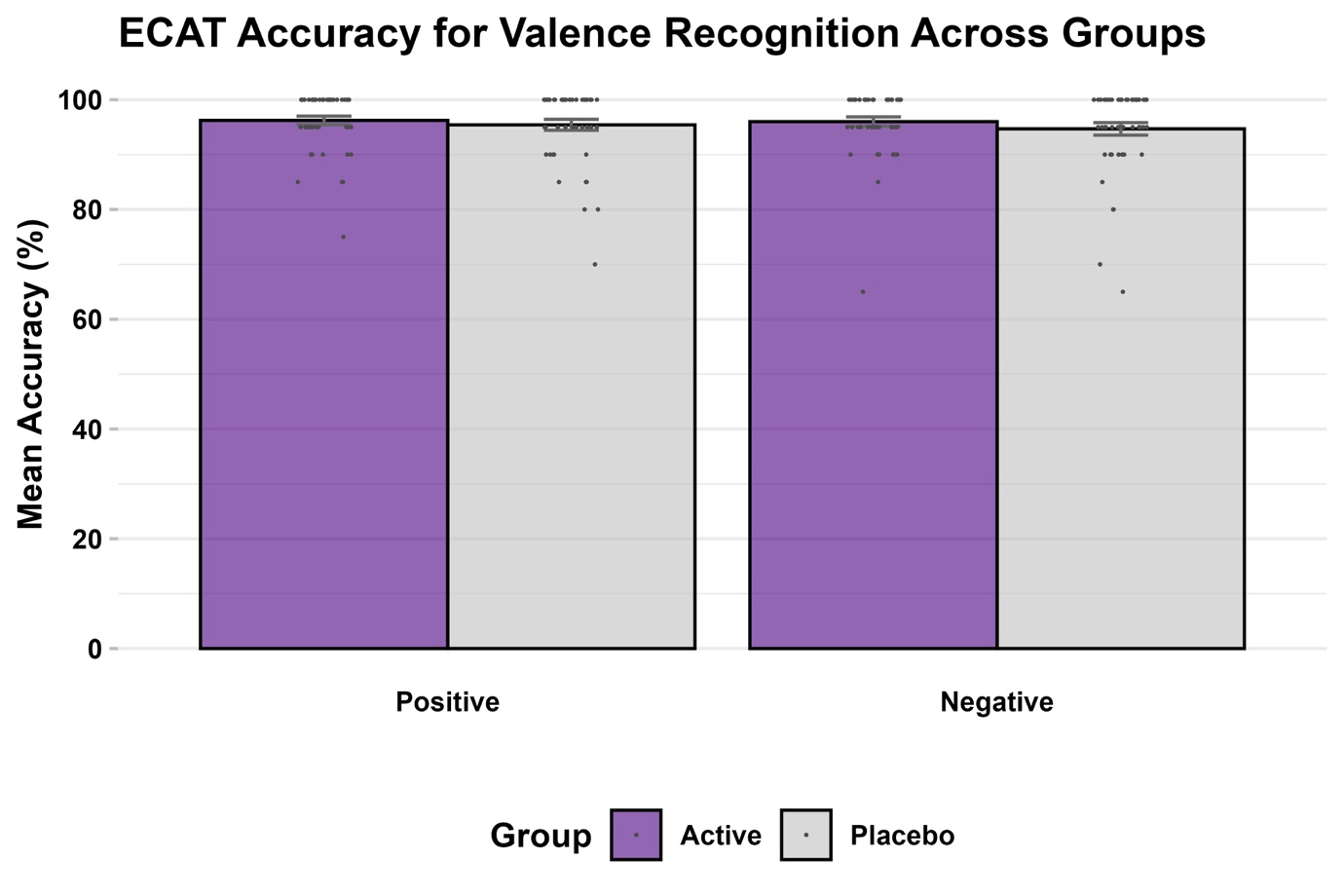

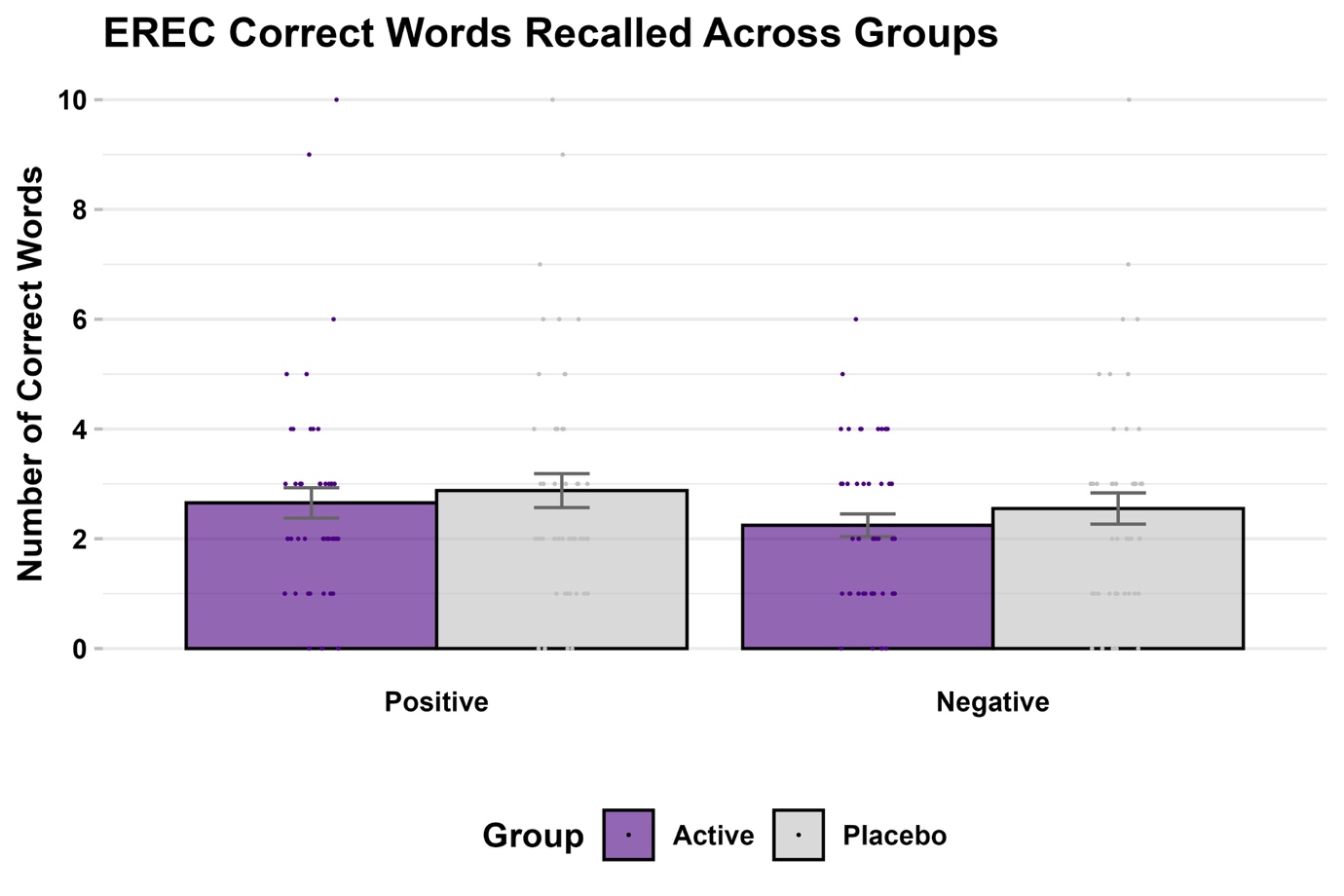

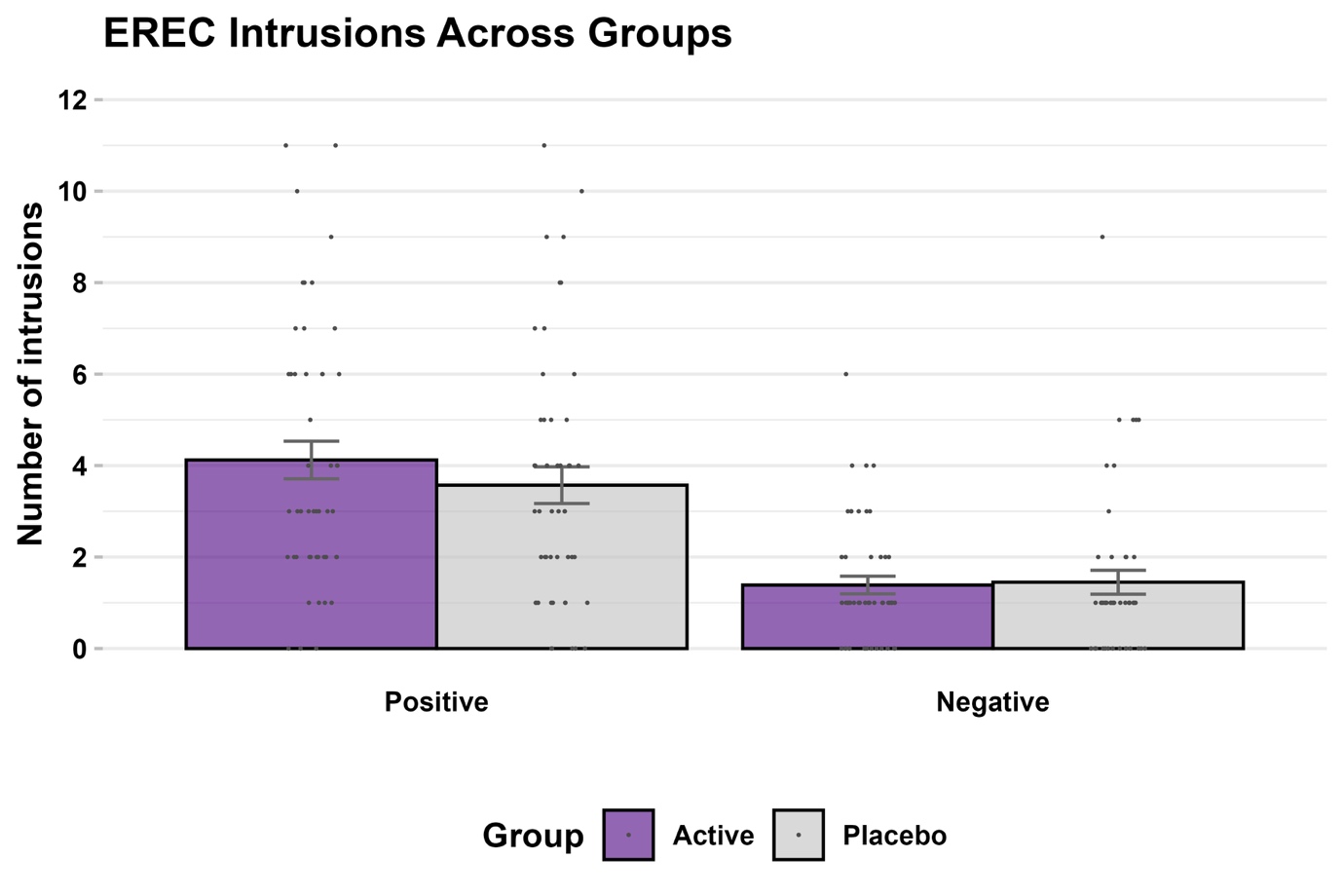

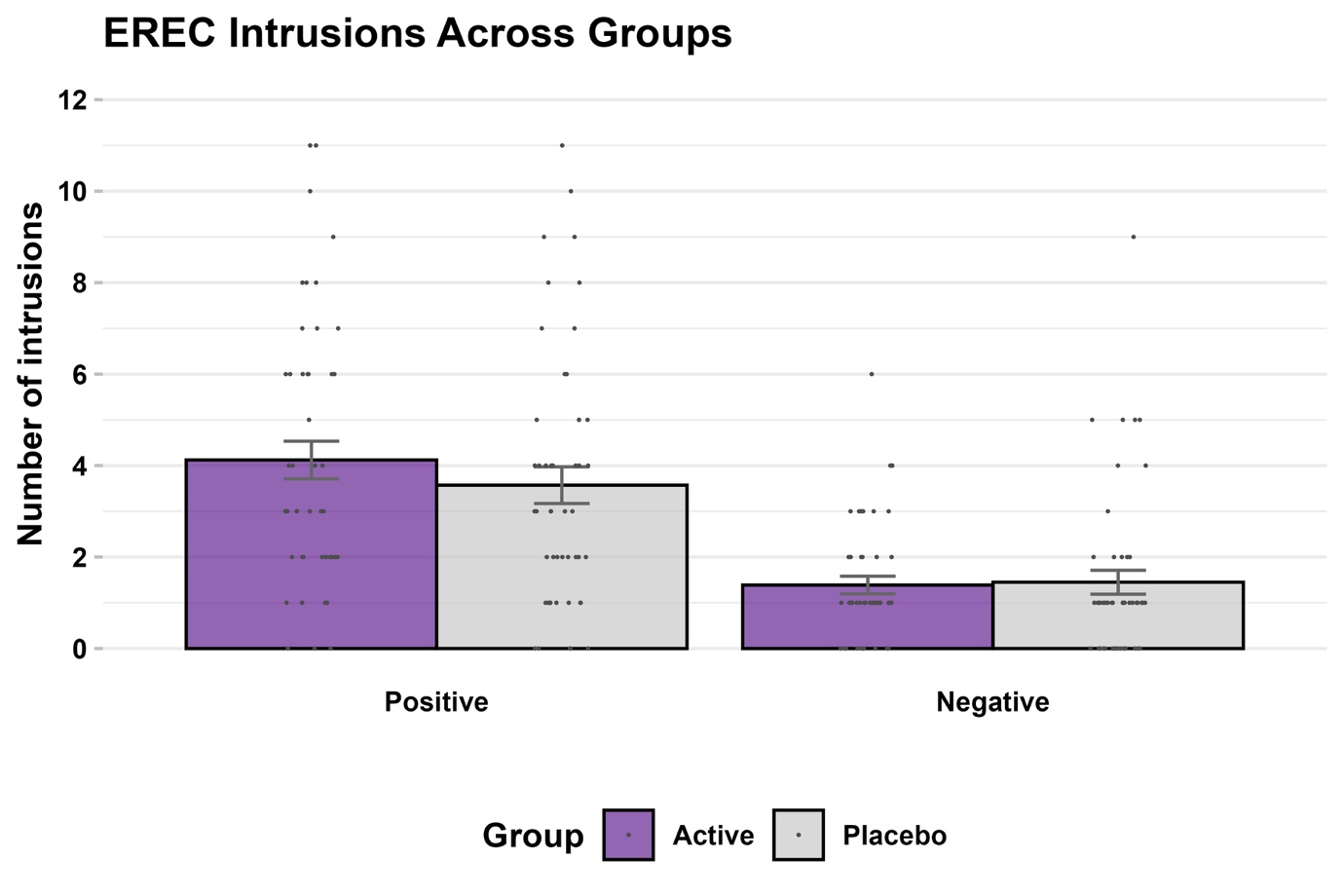

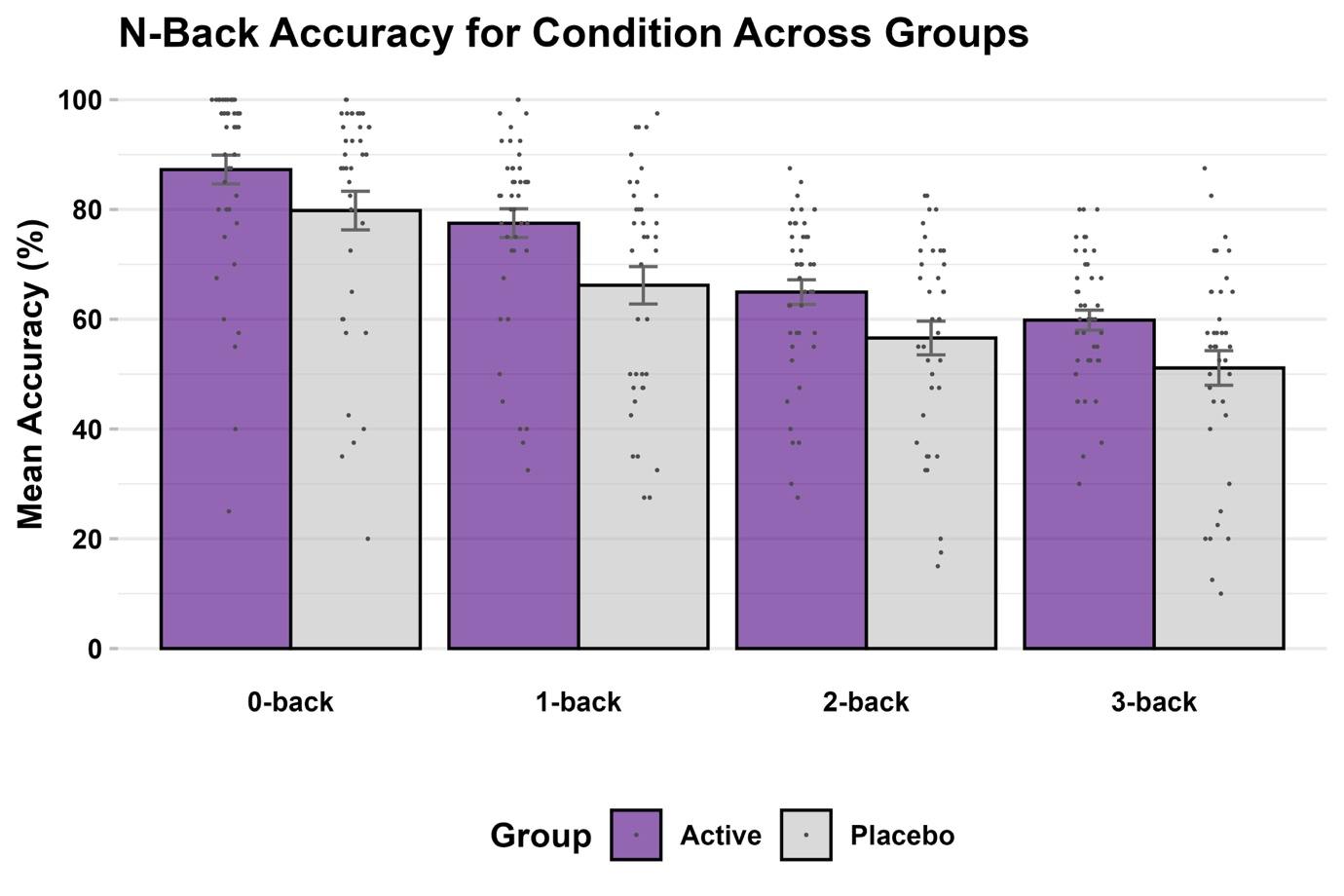

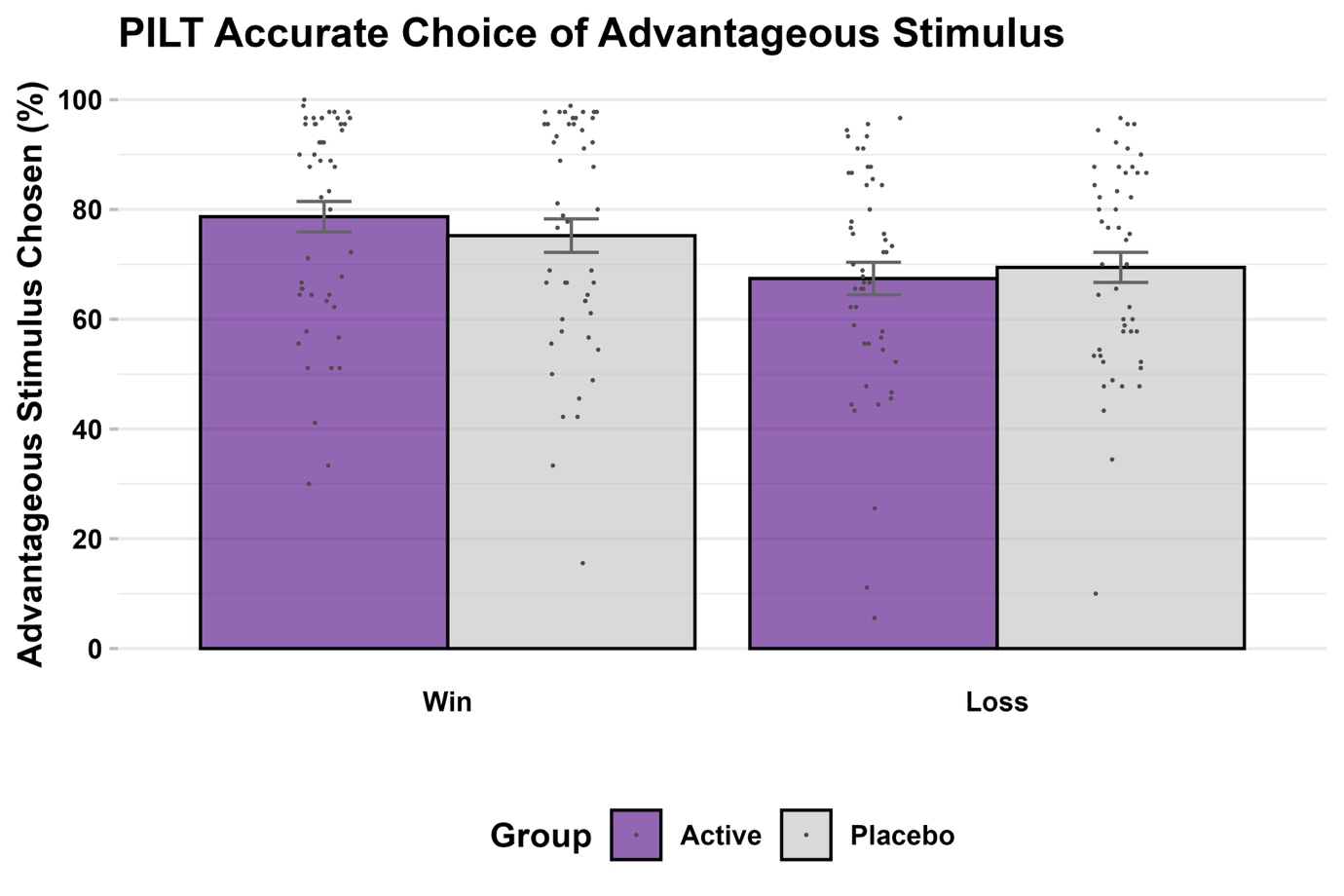

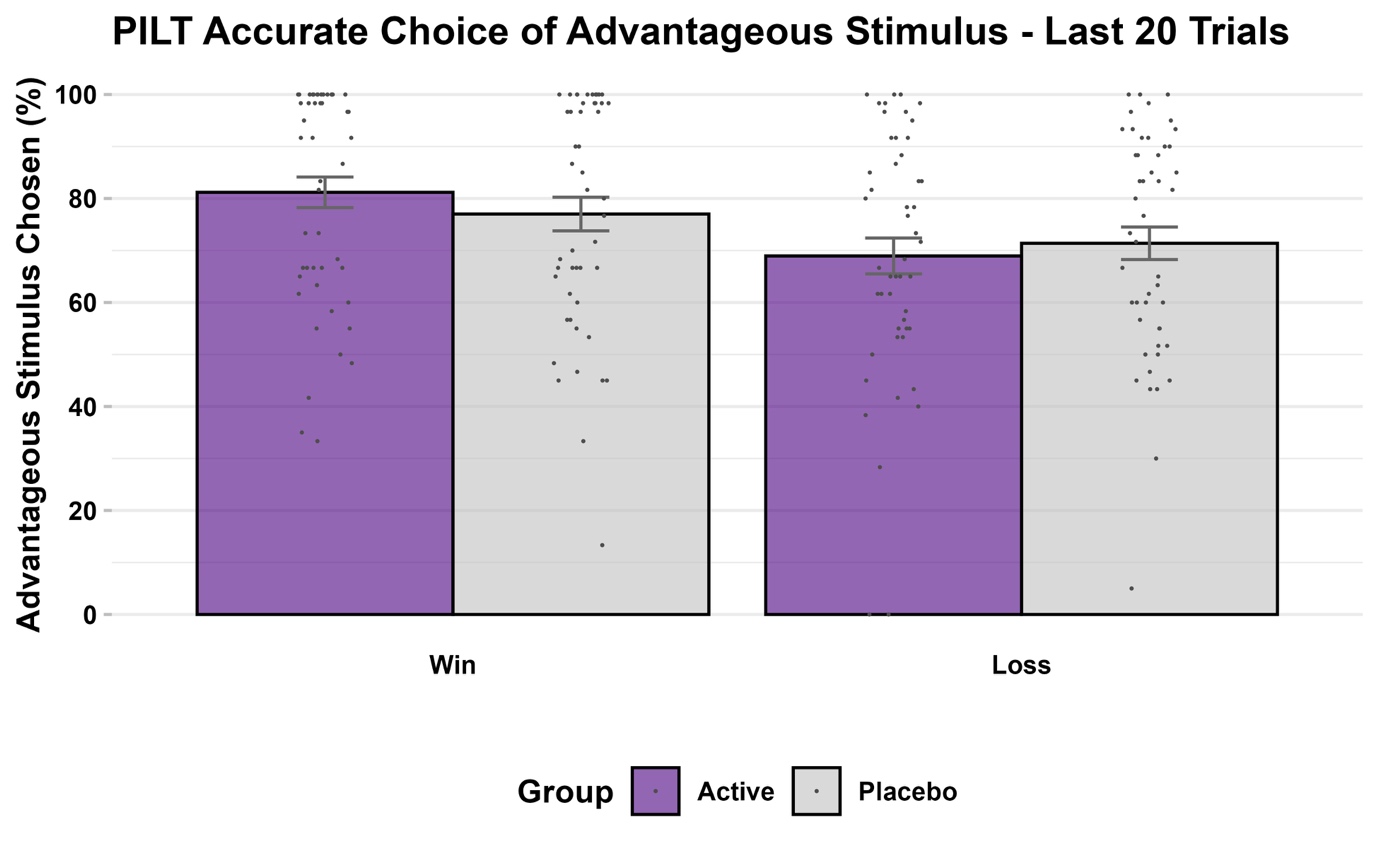


***Supplementary Material, S8.*** *Post-hoc Bayesian mixed-effects modelling for the FERT accuracy data (i.e., primary outcome)*

The results of the *post-hoc* Bayesian mixed-effects modelling were consistent with those of frequentist-based modelling (i.e., repeated measure ANOVA) for the FERT accuracy data (Table S8.1). The effects of each model were considered significant if the 95% credible interval did not include zero.

*Table S8.1.* Bayesian Mixed Effects Modelling Results

|  | *Estimate* | *Est. Error* | *95% CI, Lower* | *95% CI, Upper* |
| --- | --- | --- | --- | --- |
| ***Main effect*** |  |  |  |  |
| Group | -0.00 | 0.02 | -0.03 | 0.03 |
| Anger | 0.18 | 0.02 | 0.15 | 0.22 |
| Disgust | 0.08 | 0.01 | 0.05 | 0.11 |
| Fear | 0.06 | 0.01 | 0.03 | 0.09 |
| Happy | 0.23 | 0.02 | 0.18 | 0.27 |
| Sad | 0.09 | 0.01 | 0.06 | 0.12 |
| Surprise | 0.18 | 0.02 | 0.14 | 0.22 |
|  |  |  |  |  |
| ***Group-Emotion interaction*** |  |  |  |  |
| Group × Anger | -0.01 | 0.02 | -0.04 | 0.03 |
| Group × Disgust | 0.01 | 0.02 | -0.02 | 0.05 |
| Group × Fear | 0.00 | 0.02 | -0.03 | 0.04 |
| Group × Happy | 0.02 | 0.02 | -0.02 | 0.05 |
| Group × Sad | -0.00 | 0.02 | -0.04 | 0.03 |
| Group × Surprise | -0.01 | 0.02 | -0.05 | 0.02 |

*Legend*: Output from mixed effects Bayesian modelling via *brms* with an expected Gaussian distribution. The factor ‘emotion’ was coded with ‘neutral’ serving as a reference variable.

The model demonstrated adequate convergence (rhat = 1.00) within a narrow confidence interval (95% CI 0.06, 0.07), and posterior predictive checks demonstrated reasonable model fit (Figure S8.1).

*Figure S8.1.* Posterior probability checks across the model predicted values (*y*_rep_) and observed values (*y*) to assess model fit.


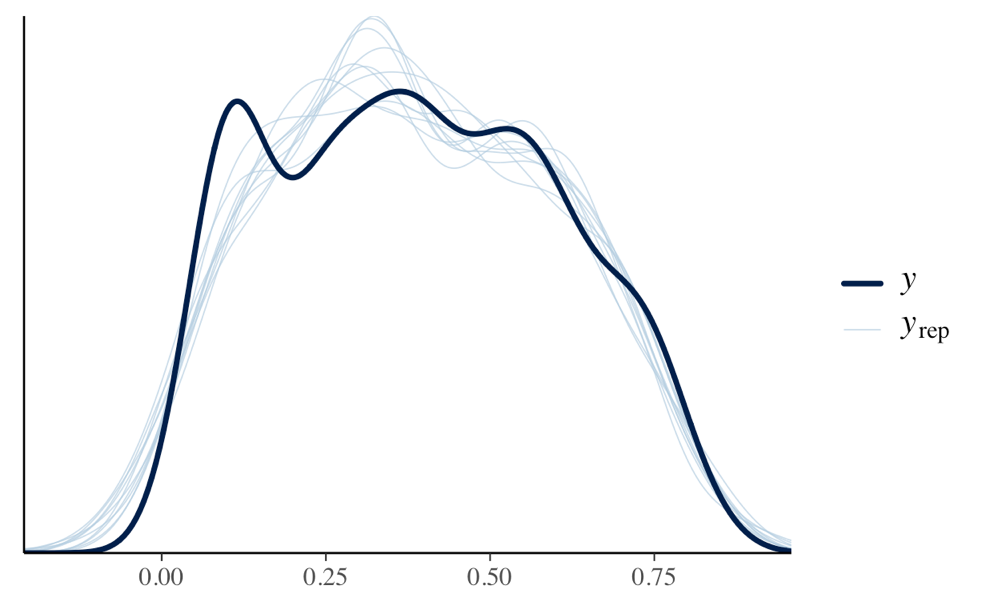


There was a significant main effect of emotion (with reference to neutral faces), while the main effect of group and the group-emotion interaction were non-significant.

Further Bayes Factor (BF) analyses were conducted to explore the strength of evidence for or against the alternative hypothesis. There was very strong evidence for the main effect of emotional expression on unbiased hit rate (BF_01_ = 2.778395e+213), while there was strong evidence that the groups had equal unbiased hit rates (BF_10_ = 0.09). Similarly, there was strong evidence for a lack of interaction between group and emotion on unbiased hit rate (BF_10_ = 0.003).

***Supplementary Material, S9.*** *Post-hoc computational reinforcement learning modelling for the PILT*

Baseline-adjusted ANCOVA models showed no statistically significant difference across groups for both learning rate (F_1,96_ = 0.09, p = 0.759) and outcome sensitivity (F_1,96_ = 0.59, p = 0.44) model parameters (see Figure S9.1).

*Figure S9.1.* Log-transformed learning rate (A) and outcome sensitivity (B) across allocation groups.


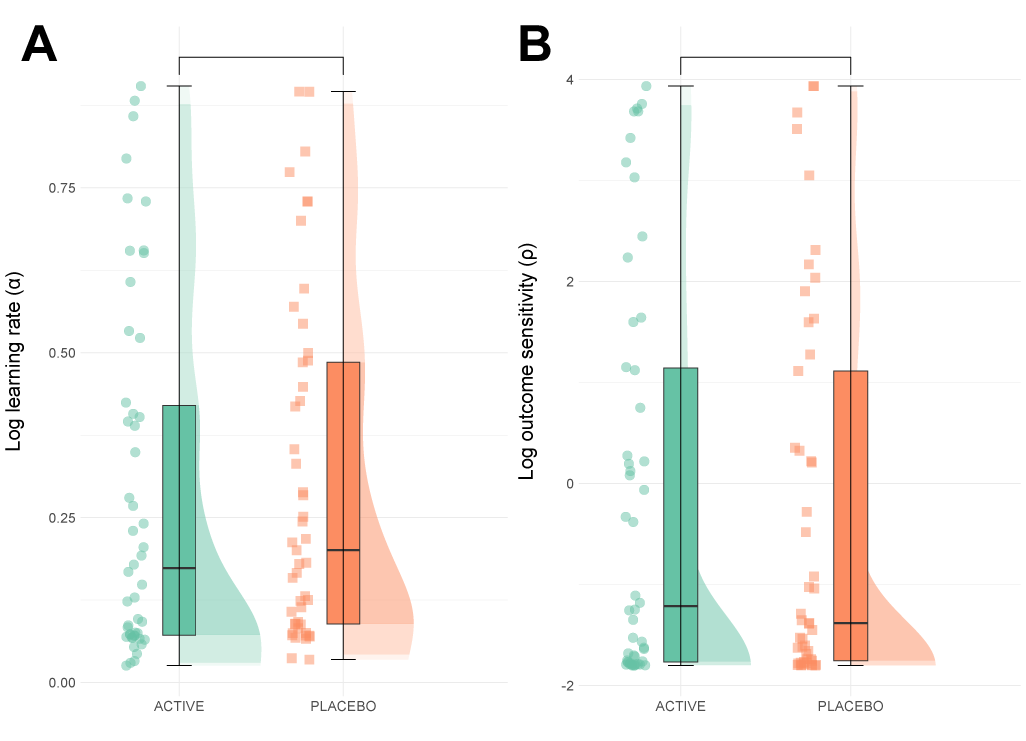


*Legend:* Boxplots represent the interquartile range (IQR), while the central line depicts the median. The whiskers extend to approximately ± 1.5 times the IQR, encompassing the bulk of the data points; half-violin plots depict the data distribution. Panels A-B contain data for N = 101 individuals.

***References***

1. Poll EM, Kreitschmann-Andermahr I, Langejuergen Y, Stanzel S, Gilsbach JM, Gressner A, et al. Saliva collection method affects predictability of serum cortisol. Clin Chim Acta. 2007;382(1-2):15-9.

2. Clements AD, Parker CR. The relationship between salivary cortisol concentrations in frozen versus mailed samples. Psychoneuroendocrinology. 1998;23(6):613-6.

3. Aardal E, Holm AC. Cortisol in saliva--reference ranges and relation to cortisol in serum. Eur J Clin Chem Clin Biochem. 1995;33(12):927-32.

4. Garde AH, Hansen AM. Long-term stability of salivary cortisol. Scand J Clin Lab Invest. 2005;65(5):433-6.

5. Harmer CJ, O'Sullivan U, Favaron E, Massey-Chase R, Ayres R, Reinecke A, et al. Effect of acute antidepressant administration on negative affective bias in depressed patients. Am J Psychiatry. 2009;166(10):1178-84.

6. Lundqvist D, Flykt A, Öhman A. Karolinska directed emotional faces. Cognition and Emotion. 1998.

7. Young AW, Rowland D, Calder AJ, Etcoff NL, Seth A, Perrett DI. Facial expression megamix: Tests of dimensional and category accounts of emotion recognition. Cognition. 1997;63(3):271-313.

8. Grier JB. Nonparametric indexes for sensitivity and bias: computing formulas. Psychological bulletin. 1971;75(6):424.

9. Anderson NH. Likableness ratings of 555 personality-trait words. Journal of personality and social psychology. 1968;9(3):272.

10. Pessiglione M, Seymour B, Flandin G, Dolan RJ, Frith CD. Dopamine-dependent prediction errors underpin reward-seeking behaviour in humans. Nature. 2006;442(7106):1042-5.

11. Owen AM, McMillan KM, Laird AR, Bullmore E. N-back working memory paradigm: A meta-analysis of normative functional neuroimaging studies. Human Brain Mapping. 2005;25(1):46-59.

12. Halahakoon DC, Kaltenboeck A, Martens M, Geddes JG, Harmer CJ, Cowen P, et al. Pramipexole Enhances Reward Learning by Preserving Value Estimates. Biol Psychiatry. 2024;95(3):286-96.

13. Colwell MJ, Tagomori H, Shang F, Cheng HI, Wigg CE, Browning M, et al. Direct serotonin release in humans shapes aversive learning and inhibition. Nat Commun. 2024;15(1):6617.

14. Murphy SE, Wright LC, Browning M, Cowen PJ, Harmer CJ. A role for 5-HT(4) receptors in human learning and memory. Psychol Med. 2020;50(16):2722-30.
